# Correspondence

**Published:** 1895-01

**Authors:** Wesley Mills

**Affiliations:** Faculty of Comparative Medicine and Veterinary Science, McGill University, Montreal


### CORRESPONDENCE.

FACULTY OF COMPARATIVE MEDICINE AND VETERINARY SCIENCE,
McGILL UNIVERSITY, MONTREAL, JANUARY, 1895.

*Editor of Journal of Comparative Med. and Veter. Archives.*

GENTLEMEN: I admire very much the candor and boldness with which the Committee of the United States Veterinary Medical Association on Intelligence and Education has set forth its views. It has uttered no uncertain sound, and the time has come for much plain speaking. It is not my intention to discuss the subject of veterinary education in general at present, but simply one aspect of it, with the committee's report on which I do not quite agree, if I understand the paragraph aright. It reads: "Moreover, the course of study should be graded. Text-books should, as a rule, be used as *reference* books [italics mine], except in such cases where it is possible to use the text-book in class, and supplement the same by lectures; but graded courses of didactic lectures, with frequent written and oral reviews, are, as a rule, best." With most of this, as with the greater part of the rest of the report, I am in accord; but the general impression is given that text-books must play a very subordinate part in the education of the student of veterinary medicine. An inquiry into the facts will reveal a state of neglect of the use of books—text-books and others—that, to my mind, is much to be regretted.

It has been seriously proposed that in the education of students of human medicine "didactic lectures" should be wholly superseded by recitations from text-books. This is extreme, and I do not agree with any such proposal; but if I had to choose between two things—recitations from good textbooks and an ordinary course of lectures, without the use of text-books at all—I would prefer the former. When a man leaves college, if he will advance, he must read as well as observe and think, and it is usually no small matter that he shall *learn* to read while at college. Speaking as a practical teacher of much experience, I know that so long as the lecturer attempts to cover the whole ground, however superficially, it is vain to expect the student to "consult" or "refer to" books. If the student can "pass" on the lectures he will not, in most instances, consult books.

I have been astounded at the very limited extent to which the students in most of the veterinary colleges of America use books at all, a fact which accounts in no small degree for the very illiterate condition of many graduates. Every man must read and ponder—gain knowledge through the eye from the printed page as well as through the ear by lectures. I hold that both, however, are necessary. Books cannot take the place of the living teacher, whose business it is to put in his own way the truths stated in text-books and other works, illustrate these by experiments and by reference to specimens, medical cases, etc., and then ascertain what is the mental condition of those before him by some sort of examination.

Where is a man to learn the use of books if not at college? I fancy the committee did not intend to discourage the use of good text-books; nevertheless, its utterance in the matter is open to the construction I have placed upon it; and if it will take the trouble to ascertain the proportion of students that regularly uses text-books, I am sure it will be amazed, and will agree with me that this state of things is one that calls for immediate remedy. I speak from a long experience of many different methods of teaching and learning.

Truly yours,

WESLEY MILLS.

---

A SCHOOL OF FARRIERY. A School of Farriery has been established in Philadelphia in connection with the Masters' and Journeymen's Horseshoers' Associations. Professors Harger and Adams, of the Veterinary Department of the University of Pennsylvania, lectured before them, on January 25th, on Anatomy and Diseases of the Foot respectively.

